# A scalar Riemann–Hilbert problem on the torus: applications to the KdV equation

**DOI:** 10.1007/s13324-022-00715-4

**Published:** 2022-08-22

**Authors:** Mateusz Piorkowski, Gerald Teschl

**Affiliations:** 1 Department of Mathematics, KU Leuven, Celestijnenlaan 200B, 3001 Leuven, Belgium; 2 Faculty of Mathematics, University of Vienna, Oskar-Morgenstern-Platz 1, 1090 Wien, Austria

**Keywords:** Riemann–Hilbert problem, KdV equation, Jacobi theta functions, Primary 35Q15, 35Q53, Secondary 30F10, 33E05

## Abstract

We take a closer look at the Riemann–Hilbert problem associated to one-gap solutions of the Korteweg–de Vries equation. To gain more insight, we reformulate it as a scalar Riemann–Hilbert problem on the torus. This enables us to derive deductively the model vector-valued and singular matrix-valued solutions in terms of Jacobi theta functions. We compare our results with those obtained in recent literature.

## Introduction

### Background

The main goal of this short note is to present an alternative approach to the existence/uniqueness results for the model Riemann–Hilbert (R–H) problem presented in [9] and the construction of a singular matrix-valued solution found in [11, Sect. 6] (see also [13, Sect. 3]). Recall, that the objective of [9] and [11] was to rigorously apply the nonlinear steepest descent method to the initial value problem for the Korteweg–de Vries (KdV) equation,

$$\begin{aligned} \partial _t q(x,t)=6q(x,t)\partial _x q(x,t)-\partial _x^3 q(x,t), \quad (x,t)\in \mathbb {R}\times \mathbb {R}_+, \end{aligned}$$

with steplike initial data $q(x,0) = q(x)$:

$$\begin{aligned} \lim _{x \rightarrow \infty } q(x) = 0, \qquad \lim _{x \rightarrow -\infty } q(x) = -c^2, \quad c > 0, \end{aligned}$$

satisfying certain smoothness conditions. For large *t*, solutions to this problem display different behaviours in three regions of the (*x*, *t*)-plane characterized by the ratio *x*/*t* (see [9, Sect. 1]). Of particular interest to us is the transition region given by $-6c^2 t< x < 4c^2 t$, where solutions asymptotically converge to a modulated elliptic wave of the type (2.4). This result was proven in [11], where an ill-posedness of the corresponding holomorphic matrix model R–H problem was found.

The ill-posedness is closely related to the fact that the R–H problem for the KdV equation is formulated as a vector-valued problem. Note that, the standard Liouville-type argument relating existence to uniqueness for matrix-valued R–H problems having jump matrices with unit determinant (see for example [18, Theorem 5.6]), cannot be generalized to the vector case in a straightforward manner. In fact, uniqueness can fail despite existence, as demonstrated in [14, Sect. 2] for the simple case of a one-soliton solution. Uniqueness was restored by assuming the symmetry condition (3.3) below.

Another feature of the KdV equation playing an important role in the present note is the relationship between finite-gap solutions and elliptic Riemann surfaces (see [1, Chapter 3]). Algebro-geometric finite-gap solutions to the KdV equation can be given explicitly in terms of Jacobi theta functions via the Its–Matveev formula [15] (see also [10]). Unsurprisingly, the solution of the corresponding model R–H problem is also expressed in terms of Jacobi theta functions. Given that these functions can be regarded as multivalued functions on an underlying Riemann surface, the natural question arises whether the model R–H problem in the plane found in [9] can be viewed as a R–H problem on a Riemann surface instead. In our simple one-gap case, that would correspond to a R–H problem on a torus.

### Outline of this work

In the next section we will recall the Its–Matveev formula for finite-gap solutions of the KdV equation together with the corresponding elliptic Riemann surface $\hat{X}$ and related quantities.

In Sect. 3 we state the one-gap KdV model R–H problem and show that it characterizes solutions to the KdV equation via its Lax pair representation. We then formulate the model R–H problem as a *scalar-valued* R–H problem on another elliptic Riemann surface *X*, which can be viewed as a double covering of $\hat{X}$. Equivalently, solutions to this problem can be characterized by quasiperiodic meromorphic functions in the complex plane (see (3.21)), leading to the explicit R–H model solution found in [9] and singular solutions similar to the one described in [11, 13] in a straightforward manner. We show that in this picture the symmetry condition from (3.3) translates to halving the period (see (3.22)), while uniqueness follows from Liouville’s Theorem.

Section 4 discusses singular matrix-valued model solutions (see [2, 11, 13]). As shown in [11], there is no regular matrix-valued model solution satisfying all the standard assumptions, hence it is necessary to drop some of them. We compare our singular matrix-valued model solution to the ones found previously. We point out that the corresponding vanishing problem has a nontrivial solution, meaning that there is no uniqueness for the associated singular model problem. In particular, the solutions described in [2, 11] and [13] differ from the one we present in Sect. 4.

## Riemann surfaces related to Its–Matveev solutions

The following section is based on the material found in [10] (see also [1] for more details). We will consider one gap solutions $q^{IM}(x,t)$ of Its–Matveev type, with spectrum given by

$$\begin{aligned} \sigma := \sigma (H) = [-c^2, -a^2] \cup \mathbb {R}_+, \qquad 0< a < c, \end{aligned}$$

where

$$\begin{aligned} H = -\partial _x^2 + q^{IM}, \qquad {{\,\mathrm{dom}\,}}(H) := H^2(\mathbb {R}) \subset L^2(\mathbb {R}), \end{aligned}$$

is the associated self-adjoint Schrödinger operator. To this end consider the elliptic Riemann surface $\hat{X}$ associated with the function

$$\begin{aligned} \mathcal {R}(\lambda ) := \sqrt{\lambda (\lambda +c^2)(\lambda +a^2)}, \end{aligned}$$

with cuts along the spectrum $\sigma$. A point on $\hat{X}$ is denoted by $p = (\lambda ,\pm )$, $\lambda \in \mathbb {C}\cup \lbrace \infty \rbrace$.

Let $\lbrace {\hat{a}}, \hat{b}\rbrace$ be a canonical basis on $\hat{X}$, where the cycle ${\hat{b}}$ surrounds the interval $[-c^2, -a^2]$ counterclockwise on the upper sheet and the cycle ${\hat{a}}$ supplements ${\hat{b}}$ by passing along the gap $[{-a}^{2}, 0]$ in the positive direction on the lower sheet and then changing the sheet. We observe that $\hat{X}$ is a compact Riemann surface of genus one, and thus has, up to multiples, a unique holomorphic differential $d \hat{\omega }$, which can be normalized such that $\int _{\hat{a}} d\hat{\omega }= 1$. We denote the integral over the $\hat{b}$ cycle by

$$\begin{aligned} \hat{\tau }:= \int _{\hat{b}} d\hat{\omega }. \end{aligned}$$

Next introduce the Riemann constant $\hat{K} = -\frac{\hat{\tau }}{2} + \frac{1}{2}$ and define the wave and frequency numbers *V* and *W* ( [17, 19]) as the ${\hat{b}}$-periods of the normalized Abelian differentials of the second kind $d\hat{\Omega }_1$ and $d\hat{\Omega }_3$ on $\hat{X}$ uniquely defined by the order of the pole at infinity

$$\begin{aligned} d\hat{\Omega }_1 = \frac{\mathrm {i}}{2\sqrt{\lambda }}(1+ O(\lambda ^{-1}))d\lambda ,  d\hat{\Omega }_3 = -\frac{3\mathrm {i}}{2}\sqrt{\lambda }(1 +O(\lambda ^{-1}))d\lambda ,\ \ \lambda \rightarrow \infty ,\end{aligned}$$

and by the normalization conditions $\int _{\hat{a}} d \Omega _{1,3} = 0$. To be precise, we set:

2.1 $$\begin{aligned} \mathrm {i}V := \int _{\hat{b}} d\hat{\Omega }_1, \qquad \mathrm {i}W := \int _{\hat{b}} d\hat{\Omega }_3. \end{aligned}$$

The explicit form of $d\hat{\Omega }_1$, which will be needed later, is given by (see [10])

2.2 $$\begin{aligned} d\hat{\Omega }_1 := \frac{\mathrm {i}}{2}\frac{\lambda - h}{\mathcal R(\lambda )} d\lambda , \end{aligned}$$

where $h := \int _{\hat{a}} \frac{\lambda \, d\lambda }{\mathcal R(\lambda )} \big ( \int _{\hat{a}} \frac{d\lambda }{\mathcal R(\lambda )} d\lambda \big )^{-1}$. Moreover, $d\hat{\Omega }_{1,3}$ change sign under the sheet exchange map $(\lambda ,\pm ) \rightarrow (\lambda , \pm )^* := (\lambda , \mp )$, see [10, Eq. 2.3].

The final ingredient we will need is the theta function $\theta _3 = \theta _3( \, \cdot \, |\, \hat{\tau })$ of $\hat{X}$ given by

2.3 $$\begin{aligned} \theta _3(z\, | \, \hat{\tau }) := \sum _{n\in \mathbb {Z}} \exp \big \lbrace \pi \mathrm {i}\hat{\tau }n^2+2\pi \mathrm {i}z n\big \rbrace . \end{aligned}$$

As $\mathrm {i}\hat{\tau }< 0$, this series is absolutely convergent for all $z \in \mathbb {C}$. We are now in the position to state the following

### Theorem 2.1

(Its, Matveev [15]) Let $\Delta \in \mathbb {R}$ be given. The function

2.4 $$\begin{aligned} q^{IM}(x,t) := -2\frac{d^2}{dx^2} \log \theta _3\Big (\frac{1}{2\pi }(V x-4 W t+ \Delta )-\hat{K} \, \Big | \, \hat{\tau }\, \Big )-a^2-c^2-2h \end{aligned}$$

defines a global solution of the KdV equation. Moreover, $\sigma (H) = [-c^2,-a^2]\cup \mathbb {R}_+$, where *H* is the unique self-adjoint realization of the differential expression $-\partial ^2_x+ q^{IM}$ in $L^2(\mathbb {R})$.

**Fig. 1:**
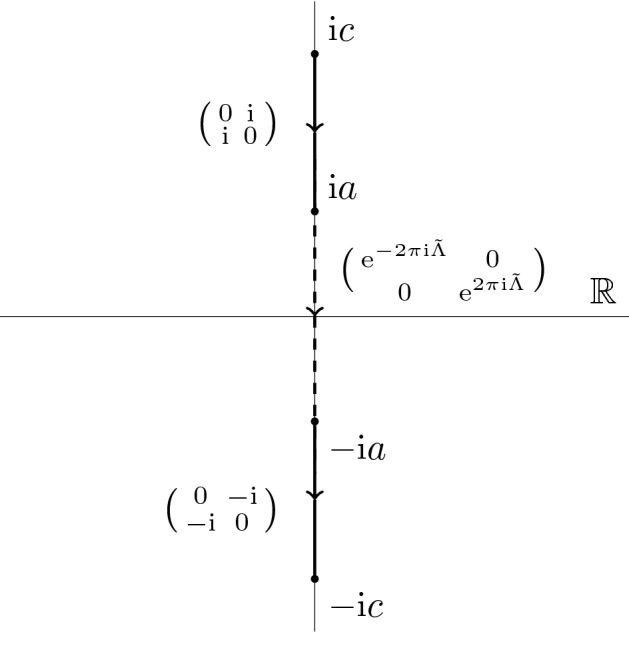
Jump contour for the model R–H problem

Similar formulas to (2.4) exist for the higher genus case, see [1, Sect. 3]. We will show in the next section that the standard vector-valued R–H problem characterizing the solution (2.4) can be formulated on an elliptic Riemann surface *X*, which will turn out to be a double covering of $\hat{X}$. The Riemann surface *X* is more suitable for the nonlinear steepest descent analysis (see [9, 13]).

## The model Riemann–Hilbert problem

In the following we recall the model vector-valued R–H problem for one-gap solutions of the KdV equation. For the underlying scattering theory and nonlinear steepest descent analysis leading to this problem in the transition region, we refer to [9, Sect. 4].

We want to find a vector-valued function $m^{\text {mod}}(k)=(m_1^{\text {mod}}(k),\ m_2^{\text {mod}}(k))$ holomorphic in the domain $\mathbb {C}\setminus [\mathrm {i}c, -\mathrm {i}c]$, continuous up to the boundary except possibly at the points $\mathcal G:=\{ \mathrm {i}c, \mathrm {i}a, -\mathrm {i}a, -\mathrm {i}c\}$ and satisfying the jump condition (see Fig. 1):

3.1 $$\begin{aligned} m_+^{\text {mod}}(k)= m_-^{\text {mod}}(k) v^{\text {mod}}(k), \end{aligned}$$

(with $m_\pm (k):=\lim _{\varepsilon \downarrow 0} m(k\pm \varepsilon )$ as usual), where

3.2 $$\begin{aligned} v^{\text {mod}}(k) = \left\{ \begin{array}{ll} \begin{pmatrix} 0 &{} \mathrm {i}\\ \mathrm {i}&{} 0 \end{pmatrix},&{} k\in [\mathrm {i}c, \mathrm {i}a], \,\\ \begin{pmatrix} 0 &{} -\mathrm {i}\\ -\mathrm {i}&{} 0 \end{pmatrix},&{} k\in [-\mathrm {i}a, -\mathrm {i}c],\\ \begin{pmatrix}\mathrm {e}^{-2\pi \mathrm {i}\tilde{\Lambda }}&{} 0\\ 0&{}\mathrm {e}^{2\pi \mathrm {i}\tilde{\Lambda }}\end{pmatrix},&{} k\in [\mathrm {i}a, -\mathrm {i}a],\\ \end{array}\right. \end{aligned}$$

the symmetry condition

3.3 $$\begin{aligned} m^{\text {mod}}(-k) = m^{\text {mod}}(k) \begin{pmatrix} 0 &{} 1 \\ 1 &{} 0 \end{pmatrix}, \end{aligned}$$

and the normalization condition

3.4 $$\begin{aligned} \lim _{k\rightarrow \mathrm {i}\infty } m^{\text {mod}}(k)= (1\ \ 1).\end{aligned}$$

At any point $\kappa \in \mathcal G$ the vector function $m^{\text {mod}}(k)$ can have at most a fourth root singularity: $m^{\text {mod}}(k)= O((k-\kappa )^{-1/4})$, $k\rightarrow \kappa$.

In Theorem 3.5 we will see that the choice $\tilde{\Lambda }= \frac{1}{2\pi }(Vx-4Wt+\Delta )$ with $\Delta \in \mathbb {R}$, will lead to the KdV solution (2.4).

### Remark 3.1

The model problem presented here, together with its higher genus generalizations, appears frequently in applications of R–H theory as the outer parametrix problem, see for example [2, Sect. 4.1], [4, Sect. 1], [5].

Of crucial importance is the following fact about solutions of the model problem.

### Proposition 3.2

For any $\tilde{\Lambda }\in \mathbb {R}$, the model R–H problem has a unique solution.

### Proof

The proof can be found in [11, Sect. 5]. However, uniqueness will follow from the material presented later in this section, see in particular Lemma 3.8. $\square$

### Elliptic Riemann surface related to the model problem

As a preparation for the proof of Theorem 3.5 on the relation between the model R–H problem and the KdV equation, we have to consider the elliptic Riemann surface *X* associated with the function

$$\begin{aligned} w(k) :=\sqrt{(k^2 + c^2)(k^2 + a^2)}, \end{aligned}$$

defined on $\mathbb {C}\setminus ([-\mathrm {i}c, -\mathrm {i}a] \cup [\mathrm {i}a, \mathrm {i}c])$ with the branch fixed by $w(0) > 0$. The two sheets of *X* are glued along the cuts $[\mathrm {i}c,\mathrm {i}a]$ and $[-\mathrm {i}a, -\mathrm {i}c]$. Points on this surface are denoted by $p=(k,\pm )$. To simplify formulas we keep the notation $k=(k,+)$ for points on the upper sheet of *X*. Clearly *w* extends to a meromorphic function on *X* via $w(p)= w(k)$ for $p=(k,+)$ and $w(p)= -w(k)$ for $p=(k,-)$.

The Riemann surface *X* is seen to be a double covering of the Riemann surface $\hat{X}$ introduced in the previous section through the mapping

3.5 $$\begin{aligned} {{\,\mathrm{Cov}\,}}:X \rightarrow \hat{X}, \quad (k, \pm ) \mapsto (k^2, \pm {{\,\mathrm{sgn}\,}}(\mathop {\mathrm {Im}}k)), \end{aligned}$$

where $\lbrace (k,\pm ) : \mathop {\mathrm {Im}}k = 0 \rbrace$ gets mapped to the cut $\mathbb {R}_+$ on $\hat{X}$. Alternatively, $\hat{X} \simeq X / \mathord {\sim }$, where $(k, +) \sim (-k,-)$. We can push forward the meromorphic differentials $d\hat{\Omega }_{1,3}$ by ${{\,\mathrm{Cov}\,}}$ to obtain meromorphic differentials $d\hat{\Omega }_{1,3}$ on *X*. In particular formula (2.2) becomes

3.6 $$\begin{aligned} d\Omega _1(k) = \mathrm {i}\frac{k^2-h}{w(k)} dk = (\mathrm {i}+ O(k^{-2})) dk. \end{aligned}$$

As in Sect. 2 we choose a canonical homology basis of cycles $\{{a}, {b}\}$ for *X* as follows: The $\mathbf{a}$-cycle surrounds the points $-\mathrm {i}a,\mathrm {i}a$ starting on the upper sheet from the left side of the cut $[\mathrm {i}c,\mathrm {i}a]$ and continues on the upper sheet to the left part of $[-\mathrm {i}a, -\mathrm {i}c]$ and returns after changing sheets. The cycle $\mathbf{b}$ surrounds the points $\mathrm {i}a, \mathrm {i}c$ counterclockwise on the upper sheet. Note that the $\mathbf{b}$-cycle is mapped via ${{\,\mathrm{Cov}\,}}$ to the $\hat{b}$-cycle on $\hat{X}$, but the image of the $\mathbf{a}$-cycle encircles the $\hat{a}$-cycle twice. Thus, the relation to the wave number and frequency remains unchanged,

$$\begin{aligned} \mathrm {i}V = \int _\mathbf{b} d\Omega _1,  \mathrm {i}W = \int _\mathbf{b} d\Omega _3, \end{aligned}$$

while the condition $\int _{\hat{a}} d \Omega _{1,3} = 0$ now reads:

$$\begin{aligned} \int _{-\mathrm {i}a}^{\mathrm {i}a} d\Omega _{1,3} = 0. \end{aligned}$$

Next we introduce the *g*-function, given by (c.f. [10, Eq. 2.17])

$$\begin{aligned} g(k) := \int _{\mathrm {i}c}^k 4\mathrm {i}\left( d\Omega _3 - \frac{\mathrm {i}x}{t} d\Omega _1 \right) , \end{aligned}$$

where we restrict the path of integration to $\mathbb {C}\setminus [-\mathrm {i}c, \mathrm {i}c]$ on the upper sheet. Note that this *g*-function is different than the one presented in [9, Lemma 4.1] in the steplike case, as it would lead to a modulated Its–Matveev solution. The following lemma holds.

#### Lemma 3.3

The function *g* satisfies the following properties:

1. *g*(*k*) is an analytic function for $k \in \mathbb {C}\setminus [-\mathrm {i}c, \mathrm {i}c]$;
2. $$g(k)=-g(-k)$$ for $$k\in \mathbb {C}\setminus [\mathrm {i}c, - \mathrm {i}c]$$;
3. $$g_-(k)+g_+(k)=0$$ as $$k\in [\mathrm {i}c, \mathrm {i}a]\cup [-\mathrm {i}a, - \mathrm {i}c]$$;
4. $$t(g_-(k) - g_+(k))=Vx-4Wt$$ as $$k\in [\mathrm {i}a, - \mathrm {i}a]$$;
5. The asymptotical behavior
  3.7 $$\begin{aligned} \Phi (k)-\mathrm {i}g(k)= O\left( \frac{1}{k}\right) \end{aligned}$$
  holds as $k\rightarrow \infty$, where we define the KdV-phase function to be
  $$\begin{aligned} \Phi (k) = \Phi (k,x,t) := 4\mathrm {i}k^3 + \mathrm {i}k \frac{x}{t}. \end{aligned}$$

Additionally, for Theorem 3.5 we will need the *x*-derivative of the $O(k^{-1})$-term in (3.7).

#### Lemma 3.4

We have

3.8 $$\begin{aligned} \int _{\mathrm {i}c}^k d\Omega _1 = \mathrm {i}k -\frac{1}{2\mathrm {i}k}(2h+a^2+c^2) + O(k^{-3}). \end{aligned}$$

#### Proof

Computing the $O(k^{-2})$-term in (3.6) leads to

$$\begin{aligned} d\Omega _1 = \left( \mathrm {i}+ \frac{1}{2\mathrm {i}k^2}(2h+a^2+c^2) + O(k^{-4})\right) dk \end{aligned}$$

and hence

$$\begin{aligned} \int _{\mathrm {i}c}^k d\Omega _1 = \mathrm {i}k + C -\frac{1}{2\mathrm {i}k}(2h+a^2+c^2) + O(k^{-3}). \end{aligned}$$

Since *g*(*k*) is odd we must have $C=0$. $\square$

In particular we have

$$\begin{aligned} t\Phi (k)-t\mathrm {i}g(k)= \frac{d(x,t)}{2k\mathrm {i}} + O(k^{-3}) \end{aligned}$$

with

$$\begin{aligned} \partial _x d(x,t) = 2h+a^2+c^2. \end{aligned}$$

The importance of the model R–H problem lies in the following

#### Theorem 3.5

Assume $\tilde{\Lambda }= \frac{1}{2\pi }(Vx-4Wt+\Delta )$, $(x,t) \in \mathbb {R}^2$, where *V* and *W* are the wave number and frequency defined in (2.1) and $\Delta$ is an arbitrary real constant. Let $m^{mod }(k,x,t)$ be the unique solution of the corresponding R–H problem, which we assume to be smooth in the variables *x* and *t*. Define

$$\begin{aligned} q^{mod }(x,t) = \partial _x Q(x,t)-2h-a^2-c^2 \end{aligned}$$

where

$$\begin{aligned} m^{mod }(k,x,t) = (1 \ \ 1) + \frac{Q(x,t)}{2k\mathrm {i}}(-1 \ \ 1) + O(k^{-2}). \end{aligned}$$

Then $q^{\text {mod}}(x,t)$ is a solution of the KdV equation.

#### Proof

First we will introduce the vector-valued function

$$\begin{aligned} \Psi (k,x,t) = \Psi (k) = m^{\text {mod}}(k) \mathrm {e}^{-\mathrm {i}tg(k)\sigma _3}, \end{aligned}$$

where $$\sigma _3 = \begin{pmatrix} 1 &{} 0 \\ 0 &{} -1 \end{pmatrix}$$ and thus $$\mathrm {e}^{-\mathrm {i}tg(k)\sigma _3} = \begin{pmatrix} \mathrm {e}^{-\mathrm {i}tg(k)} &{} 0 \\ 0 &{} \mathrm {e}^{\mathrm {i}tg(k)} \end{pmatrix}$$. Then $\Psi$ will satisfy the jump condition

3.9 $$\begin{aligned} \Psi _+(k) = \Psi _-(k) v^\Delta (k), \end{aligned}$$

where

$$\begin{aligned} v^{\Delta }(k) = \left\{ \begin{array}{ll} \begin{pmatrix} 0 &{} \mathrm {i}\\ \mathrm {i}&{} 0 \end{pmatrix},&{} k\in [\mathrm {i}c, \mathrm {i}a], \,\\ \begin{pmatrix} 0 &{} -\mathrm {i}\\ -\mathrm {i}&{} 0 \end{pmatrix},&{} k\in [-\mathrm {i}a, -\mathrm {i}c],\\ \begin{pmatrix}\mathrm {e}^{-\mathrm {i}\Delta }&{} 0\\ 0&{}\mathrm {e}^{\mathrm {i}\Delta }\end{pmatrix},&{} k\in [\mathrm {i}a, -\mathrm {i}a],\\ \end{array} \right. \end{aligned}$$

the symmetry condition

3.10 $$\begin{aligned} \Psi (-k) = \Psi (k) \begin{pmatrix} 0 &{} 1 \\ 1 &{} 0 \end{pmatrix}, \end{aligned}$$

the normalization condition

$$\begin{aligned} \Psi (k) = \big [(1 \ \ 1) + \frac{Q-d}{2k\mathrm {i}}(-1 \ \ 1) + O(k^{-2})\big ] \mathrm {e}^{-t\Phi (k)\sigma _3}, \end{aligned}$$

as $k \rightarrow \infty$, and has at most fourth root singularities for $k \rightarrow \kappa \in \mathcal G$.

Next introduce the Lax pair *L*, *P* given by

$$\begin{aligned} L = -\partial _x^2 + V, \qquad P = -4\partial _x^3+ 6V\partial _x + 3(\partial _x V) \end{aligned}$$

for some smooth potential $V = V(x,t)$ to be determined. Note that $L\Psi -k^2\Psi$ satisfies the jump condition (3.9), as the jump matrices are constant, and the symmetry condition (3.10). For $k\rightarrow \infty$ one obtains

$$\begin{aligned} L\Psi (k)-k^2\Psi (k) = \big [\big (V-\partial _x(Q-d)\big ) (1 \ \ 1) + O(k^{-1})\big ] \mathrm {e}^{-t\Phi (k) \sigma _3}. \end{aligned}$$

Choosing

3.11 $$\begin{aligned} V := \partial _x(Q-d), \end{aligned}$$

we see that $L\Psi (k)-k^2\Psi (k) = O(k^{-1}) \mathrm {e}^{-t\Phi (k) \sigma _3}$, implying that $m_0(k) := (L\Psi (k)-k^2\Phi (k))\mathrm {e}^{\mathrm {i}tg(k)\sigma _3}$ would be a symmetric vanishing solution of the model problem. However, as the model problem has a unique symmetric solution by Proposition 3.2, we can conclude $m_0 \equiv 0$ implying

3.12 $$\begin{aligned} L\Psi = k^2\Psi . \end{aligned}$$

Moreover, (3.12) implies that $\Psi$ has the form

3.13 $$\begin{aligned} \Psi (k) = \big [(1 \ \ 1) + \frac{Q-d}{2k\mathrm {i}}(-1 \ \ 1) + \frac{R}{(2k\mathrm {i})^2}(1 \ \ 1)+O(k^{-3})\big ] \mathrm {e}^{-t\Phi (k)\sigma _3}, \end{aligned}$$

where $$\partial _x R = [\partial _x(Q-d)](Q-d)+\partial _{xx}(Q-d)$$.

Using (3.11) and (3.13) we see, as before, that $(P\Psi - \partial _t \Psi )\mathrm {e}^{\mathrm {i}tg(k)\sigma _3}$ is a symmetric vanishing solution of the model problem implying

$$\begin{aligned} P\Psi = \partial _t \Psi . \end{aligned}$$

Now a standard computation shows that

$$\begin{aligned} \underbrace{\big (\partial _t L - [P,L] \big )}_{\partial _t V - 6V\partial _x V + \partial _{xxx} V}\Psi = 0. \end{aligned}$$

As $\Psi$ is nonvanishing, this implies that $V = \partial _x(Q-d) = \partial _x Q -2h-a^2-c^2$ is a solution to the KdV equation. $\square$

#### Remark 3.6

It has been proven in [10, Theorem 2.4] that $q^{mod } = \partial _x Q -2h-a^2-c^2$ is equal to the Its–Matveev solution (2.4).

### Construction of the solution to the model problem

The solution to the model R–H problem for $m^{\text {mod}}(k)$ was given in [9, Sect. 5]. As mentioned in the introduction, we want to solve this problem in a different way, which should shed some further light on the model problem. For this, it will be convenient to denote by *m*(*k*) a generic vector-valued meromorphic function satisfying the jump condition (3.2).

For our first transformation we define

$$\begin{aligned} \tilde{\gamma }(k) = \root 4 \of {\frac{k^2+a^2}{k^2+c^2}} \end{aligned}$$

with the branch cuts along $[\pm \mathrm {i}a, \pm \mathrm {i}c]$ and the branch chosen such that $\tilde{\gamma }(k)>0$ for $k\in [\mathrm {i}c, \mathrm {i}\infty )$. Note that we have $\tilde{\gamma }(-k)=\tilde{\gamma }(k)$ and $\tilde{\gamma }(k)>0$ for $k\in \mathbb {R}$. Then $\tilde{\gamma }(k)$ solves the scalar R–H problem

$$\begin{aligned} \tilde{\gamma }_+(k)&= \pm \mathrm {i}\tilde{\gamma }_-(k), \qquad k\in [\pm \mathrm {i}a, \pm \mathrm {i}c], \\ \lim _{k\rightarrow \infty } \tilde{\gamma }(k)&= 1, \end{aligned}$$

and we set

3.14 $$\begin{aligned} m(k) = \tilde{\gamma }(k) n(k) \end{aligned}$$

such that *n*(*k*) satisfies the jump condition (3.2), except that the jumps on $[-\mathrm {i}c,-\mathrm {i}a]$ and $[\mathrm {i}c,\mathrm {i}a]$ are replaced by

3.15 $$\begin{aligned} n_+(k) = n_-(k) \begin{pmatrix} 0 &{} 1 \\ 1 &{} 0 \end{pmatrix}. \end{aligned}$$

In this setup, the two components $n_1$, $n_2$ of the vector $n :\mathbb {C}\setminus [-\mathrm {i}c,\mathrm {i}c] \rightarrow \mathbb {C}^2$ can be regarded as the values of a single function $N :X \rightarrow \mathbb {C}$ on the upper, lower sheet, respectively. Explicitly,

3.16 $$\begin{aligned} n(k) = (N((k,+)),N((k,-))). \end{aligned}$$

In this case the jump condition (3.15) implies that *N* will have no jump along the cuts, where the two sheets are glued together. However, the other jump will remain. In fact, the jump contour on *X* is a closed loop passing through the two branch points $-\mathrm {i}a$ and $\mathrm {i}a$ (from $\mathrm {i}a$ along the upper sheet to $-\mathrm {i}a$ and back to $\mathrm {i}a$ on the lower sheet), on which we have the jump condition

3.17 $$\begin{aligned} N_+(p) = N_-(p) \mathrm {e}^{-2\pi \mathrm {i}\tilde{\Lambda }}. \end{aligned}$$

Note that the symmetry condition (3.3) translates to

3.18 $$\begin{aligned} N(p^*) = N(-p), \end{aligned}$$

where $(k,\pm )^* = (k,\mp )$ denotes the sheet exchange map and $-(k,\pm )=(-k,\pm )$.

The normalized holomorphic differential is given by

$$\begin{aligned} d\omega =\Gamma \frac{d\zeta }{w(\zeta )}, \quad \text {where}\ \Gamma :=\left( \int _\mathbf{a} \frac{d \zeta }{w(\zeta )}\right) ^{-1}\in \mathrm {i}\mathbb {R}_-, \end{aligned}$$

such that $\int _\mathbf{a}d\omega =1$ and

$$\begin{aligned} \tau =\int _\mathbf{b} d\omega \in \mathrm {i}\mathbb {R}_+. \end{aligned}$$

One can check that $\hat{\tau }= 2\tau$.

In the following we will make use of certain algebraic properties of $\theta _3$ introduced in Eq. (2.3), see for example [3]. Recall that $\theta _3$ is even, $\theta _3(-z\,\big |\,\tau )=\theta _3(z\,\big |\,\tau )$, and satisfies

3.19 $$\begin{aligned} \theta _3(z+ n + \tau \ell \,\big |\,\tau )=\theta _3(z\,\big |\,\tau )\mathrm {e}^{- \pi \mathrm {i}\tau \ell ^2 - 2\pi \mathrm {i}\ell z} \quad \text {for}\quad \ell ,n \in \mathbb {Z}. \end{aligned}$$

Furthermore, let $A(p)=\int _{\mathrm {i}c}^p d\omega$ be the Abel map [12, Sect. III.6] on *X*. We identify the upper sheet of *X* with the complex plane $\mathbb {C}\setminus ([\mathrm {i}c, \mathrm {i}a]\cup [-\mathrm {i}a, -\mathrm {i}c])$. Restricting the path of integration to $\mathbb {C}\setminus [\mathrm {i}c, -\mathrm {i}c]$ we observe that *A*(*k*) is a holomorphic function in that given domain with the following properties:

- $$A_+(k)=-A_-(k) \pmod {1}$$, for $$k\in [\mathrm {i}c, \mathrm {i}a]\cup [-\mathrm {i}a, -\mathrm {i}c]$$;
- $$A_+(k)-A_-(k)=-\tau $$, for $$k\in [\mathrm {i}a, -\mathrm {i}a]$$;
- $$A(-k)=-A(k) + \frac{1}{2}$$, for $$k\in \mathbb {C}\setminus [\mathrm {i}c, -\mathrm {i}c]$$,
- $$A_+(\mathrm {i}a)=-\frac{\tau }{2} =-A_-(\mathrm {i}a)$$, $$A_+(-\mathrm {i}a) = -\frac{\tau }{2} + \frac{1}{2}$$, $$A_-(-\mathrm {i}a)=\frac{\tau }{2} + \frac{1}{2}$$.
- $$A(\infty )=\frac{1}{4}$$, $$A(k)= \frac{1}{4} -\Gamma k^{-1} + O(k^{-3})$$, as $$k\rightarrow \infty $$.

For points on the lower sheet we set $A(p^*)=-A(p)$. Finally, denote by $K= \frac{1+\tau }{2}$ the Riemann constant associated with *X* and abbreviate $\infty _\pm = (\infty ,\pm )$, $0_\pm = (0,\pm )$. Note that $A(0_+)=\frac{1}{4}+\frac{\tau }{2}$. By Riemann’s vanishing theorem [12, Sect. VI.3] the zeros of $\theta _3$ are simple and given by $z = K +\mathbb {Z}+\tau \mathbb {Z}$.

**Fig. 2:**
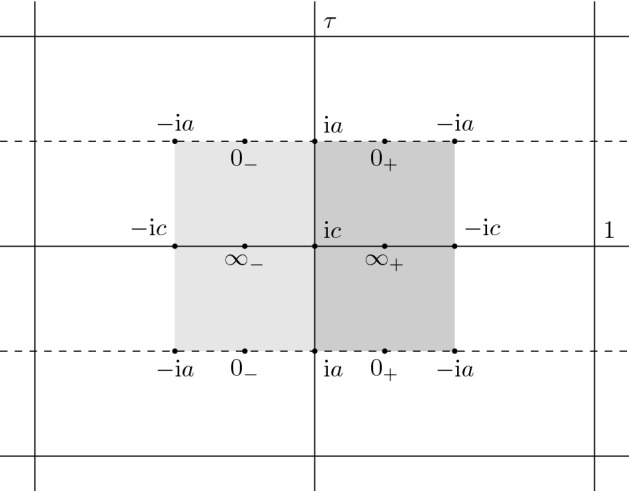
Images of important points on the Jacobi variety (dark/light gray denotes the upper/lower sheet)

According to the Jacobi inversion theorem [12, Sect. III.6], the Abel map *A* maps our Riemann surface *X* bijectively to its associated Jacobi variety $\mathbb {C}/ (\mathbb {Z}+\tau \mathbb {Z})$ depicted in Fig. 2.

The jump contour is indicated by the dashed line, while the dark/light shaded region correspond to the upper/lower sheet. Moreover, if we set

3.20 $$\begin{aligned} E(A(p))=N(p) \end{aligned}$$

for some meromorphic function *E*(*z*), then *N* will satisfy our original jump condition (3.17) if and only if

3.21 $$\begin{aligned} E(z+1) = E(z), \qquad E(z+\tau ) = E(z)\mathrm {e}^{2\pi \mathrm {i}\tilde{\Lambda }}, \end{aligned}$$

and it will satisfy the symmetry condition if and only if

3.22 $$\begin{aligned} E(z+\tfrac{1}{2}) = E(z). \end{aligned}$$

If this latter condition holds we will call *E* symmetric. If we have $E(z+\frac{1}{2}) = -E(z)$, we will call *E* anti-symmetric.

At this stage we remind the reader that we have four equivalent ways of describing vector-valued functions satisfying the jump condition (3.2):

$$\begin{aligned} m(k) \quad \longleftrightarrow \quad n(k) \quad \longleftrightarrow \quad N(p) \quad \longleftrightarrow \quad E(z) \end{aligned}$$

related via (3.14), (3.16) and (3.20) respectively. The most convenient framework will be given through the quasiperiodic meromorphic functions *E*(*z*) characterized by (3.21). Let us consider the space $\mathcal {F}(\tilde{\Lambda })$ of all quasiperiodic meromorphic functions on $\mathbb {C}/ (\mathbb {Z}+\tau \mathbb {Z})$ satisfying (3.21), without imposing any symmetry requirements. Note that $E\in \mathcal {F}(\tilde{\Lambda })$ is uniquely determined up to a constant by its divisor $(E)= \sum _{j=1}^n \mathcal {D}_{z_j}- \sum _{j=1}^n \mathcal {D}_{p_j}$, since the quotient of two such functions with the same divisor is elliptic (i.e., doubly periodic with periods 1 and $\tau$) without poles, hence a constant. Moreover, since $\frac{E'}{E}$ is elliptic, integrating this function along a fundamental domain (i.e. a parallelogram generated by 1 and $\tau$) shows that the number of zeros and poles must be equal. Note also that there must be at least one pole (unless $\tilde{\Lambda }=0$). Integrating $z\frac{E'(z)}{E(z)}$ along a fundamental domain gives

3.23 $$\begin{aligned} \sum _{j=1}^n z_j - \sum _{j=1}^n p_j = \tilde{\Lambda } \pmod {\mathbb {Z}+\tau \mathbb {Z}}. \end{aligned}$$

Choosing representatives $z_j$, $p_j \in \mathbb {C}$ such that

3.24 $$\begin{aligned} \sum _{j=1}^n z_j - \sum _{j=1}^n p_j = \tilde{\Lambda } \pmod {\mathbb {Z}}, \end{aligned}$$

we can represent *E* as

3.25 $$\begin{aligned} E(z) = E_0\prod _{j=1}^n \frac{\theta _3(z-z_j-K|\tau )}{\theta _3(z-p_j-K|\tau )}. \end{aligned}$$

Indeed the right-hand side has the required zeros and poles while (3.19) and (3.24) ensure that it satisfies the quasiperiodicity conditions (3.21).

#### Lemma 3.7

The divisor of $E\in \mathcal {F}(\tilde{\Lambda })$ is invariant with respect to translations of $\frac{1}{2}$ if and only if *E* is either symmetric or anti-symmetric

#### Proof

Observe that $C=\frac{E(z+\frac{1}{2})}{E(z)}$ is elliptic without poles and hence constant. Moreover, $C^2=\frac{E(z+\frac{1}{2})}{E(z)} \cdot \frac{E(z+1)}{E(z+\frac{1}{2})} =1$ shows $C=\pm 1$. The converse is trivial. $\square$

#### Lemma 3.8

If $E\in \mathcal {F}(\tilde{\Lambda })$ is (anti-)symmetric and has at most two poles, it is already uniquely determined up to a constant by its poles. Conversely, for each choice of two poles $p_1$, $p_2=p_1+\frac{1}{2}$ there is a unique (up to constants) symmetric and a unique anti-symmetric function $E\in \mathcal {F}(\tilde{\Lambda })$, with at most simple poles at $p_1$ and $p_2$. In fact $p_1$, $p_2$ are simple poles, unless $\tilde{\Lambda }\in \mathbb {Z}$, in which case the symmetric solution is constant.

#### Proof

Let *E* be (anti-)symmetric and nonconstant. Denote its poles by $p_1$, $p_2=p_1+\frac{1}{2} \pmod {\mathbb {Z}+\tau \mathbb {Z}}$ and its zeros by $z_1$, $z_2=z_1+\frac{1}{2} \pmod {\mathbb {Z}+\tau \mathbb {Z}}$. Choosing representatives in $\mathbb {C}$, (3.23) implies $2(z_1- p_1) = \tilde{\Lambda }+ \ell +n \tau$ for some $\ell ,n\in \mathbb {Z}$. In particular, since adding a period to $z_1$ is irrelevant, we can assume $\ell ,n\in \{0,1\}$. If $\ell =1$ this just amounts to exchanging $z_1$ and $z_2$ and hence we can assume $\ell =0$ without loss of generality. Now using $z_1 = p_1 + \frac{\tilde{\Lambda }}{2} + n\frac{\tau }{2}$, we can set $z_2= z_1 + \frac{1}{2} - n \tau$ and $p_2 = p_1 + \frac{1}{2}$ such that (3.24) holds. Now one computes using (3.19) that (3.25) fulfills $E(z+\frac{1}{2})=(-1)^n E(z)$. In other words, $p_1 \in \mathbb {C}/(\mathbb {Z}+\tau \mathbb {Z})$ and $n \in \mathbb {Z}_2$ uniquely determine *E* up to a constant. One can check, that the zeros and poles cancel if and only if $n = 0$ and $\tilde{\Lambda }\in \mathbb {Z}$, corresponding to a constant symmetric solution. $\square$

#### Corollary 3.9

If $E\in \mathcal {F}(\tilde{\Lambda })$ has at most two poles $p_1$, $p_2=p_1+\frac{1}{2}$ then there exist unique $c_s,c_a \in \mathbb {C}$ such that $E=c_s E_s + c_a E_a$, where $E_s$, $E_a$ are the symmetric, anti-symmetric solutions constructed in the previous lemma, respectively.

Returning to our original model problem, we want the poles of *E* to lie at the images of $\mathrm {i}a$ and $-\mathrm {i}a$ under the Abel map *A*, that is $p_1=\frac{\tau }{2}$ and $p_2=\frac{1+\tau }{2} = K$. The reason is that we require $m^{\text {mod}}(k)$ to be holomorphic, with at most fourth root singularities at points of $\mathcal G$. As $\tilde{\gamma }(k)$ has fourth root zeros at $\pm \mathrm {i}a$ and the Abel map *A* has square root singularities at the points of $\mathcal G$, simple poles at $\frac{\tau }{2}$, $\frac{1+\tau }{2}$ in $\mathbb {C}/(\mathbb {Z}+\tau \mathbb {Z})$ translate to fourth root singularities at $\pm \mathrm {i}a$ of $m^{\text {mod}}(k)$ under the inverse of the Abel map. In fact, this is the only choice of the pole structure leading to a $m^{\text {mod}}(k)$ holomorphic away from the jump contour with at most fourth root singularities at the contour.

For the zeros of the symmetric ($n = 0$) and the anti-symmetric ($n = 1$) solution we use $z_1 = \frac{\tilde{\Lambda }}{2} + \frac{(n+1) \tau }{2}$, $z_2= \frac{\tilde{\Lambda }}{2} + \frac{1-(n-1)\tau }{2}$. Denote by

$$\begin{aligned} E_s(z)= \frac{\theta _3(z-\frac{\tilde{\Lambda }}{2}+\frac{1}{2}|\tau )\theta _3(z-\frac{\tilde{\Lambda }}{2}|\tau )}{\theta _3(z+\frac{1}{2}|\tau )\theta _3(z|\tau )} \end{aligned}$$

the corresponding symmetric and by

$$\begin{aligned} E_a(z)= \frac{\theta _3(z-\frac{\tilde{\Lambda }}{2}-\frac{1+\tau }{2}|\tau )\theta _3(z-\frac{\tilde{\Lambda }}{2}+\frac{\tau }{2}|\tau )}{\theta _3(z+\frac{1}{2}|\tau )\theta _3(z|\tau )} \end{aligned}$$

the corresponding anti-symmetric solution. Using the identity (cf. [8] formula (1.4.3))

$$\begin{aligned} \textstyle \theta _3(z|\tau ) \theta _3(z+ \frac{1}{2} |\tau ) = \theta _3(2z+\frac{1}{2} | 2\tau )\,\theta _3(\frac{1}{2} | 2\tau ), \end{aligned}$$

(note that the quotient of both sides is a holomorphic elliptic function which equals 1 at $z=\frac{1}{2}$) we can write the formula for $E_s$ somewhat more compactly as

3.26 $$\begin{aligned} E_s(z) = \frac{\theta _3(2z-\tilde{\Lambda }+ \frac{1}{2}|2\tau )}{\theta _3(2z+\frac{1}{2}|2\tau )}. \end{aligned}$$

This is the analytic counterpart to the observation (3.22), which states that the symmetry condition is related to halving of the (real) period, or equivalently to doubling of the half-period ratio $\tau$. Indeed, $E_s$ can be viewed as a function on the Riemann surface obtained by just taking one copy of $\mathbb {C} \setminus ([-\mathrm {i}a, -\mathrm {i}c]\cup [\mathrm {i}c, \mathrm {i}a])$ and gluing the two cuts together. The resulting Riemann surface has period $2\tau = \hat{\tau }$ and is thus biholomorphically equivalent to $\hat{X}$ defined in Sect. 2. More explicitly we have that the covering map (3.5), viewed as a map between Jacobi varieties, takes on the simple form

$$\begin{aligned} {{\,\mathrm{Cov}\,}}:X \simeq \mathbb {C}/ (\mathbb {Z}+ \tau \mathbb {Z}) \rightarrow \hat{X} \simeq \mathbb {C}/ (\mathbb {Z}+ \hat{\tau }\mathbb {Z}), \qquad z \mapsto 2z. \end{aligned}$$

We see that for the symmetric case $n=0$, we have $\mathrm {i}\mathop {\mathrm {Im}}(z_j) = \frac{\tau }{2} \pmod {\mathbb {Z}+\tau \mathbb {Z}}$ and both zeros will be on $[-\mathrm {i}a, \mathrm {i}a]$ (see Fig. 2). In the anti-symmetric case $n=1$ we have $\mathrm {i}\mathop {\mathrm {Im}}(z_j) =0 \pmod {\mathbb {Z}+\tau \mathbb {Z}}$ and both zeros will be on $(\infty ,-\mathrm {i}c] \cup [\mathrm {i}c, \infty ]$. In particular, if $\tilde{\Lambda }=\frac{1}{2} \pmod {1}$ the two zeros of the anti-symmetric solution will be at $\infty _\pm$ and we cannot normalize at this point. Moreover, if $\tilde{\Lambda }=0 \pmod {1}$ such that we are looking for elliptic functions, we have $E_s(z)=1$ (i.e. zeros and poles coincide) and the zeros of $E_a(z)$ will be at $z_1=0$ and $z_2=\frac{1}{2}+\tau$.

From Corollary 3.9 it follows that all solutions of (3.17) with poles at most at $\pm \mathrm {i}a$ are given by

$$\begin{aligned} N(p) \!=\! c_s N_s(p) + c_a N_a(p), \quad N_s(p)\!=\!E_s(A(p)), \ N_a(p)\!=\!E_a(A(p)), \quad c_s,c_a \!\in \! \mathbb {C}, \end{aligned}$$

and we have

$$\begin{aligned} N(\infty _\pm )= c_s E_s(\tfrac{1}{4}) \pm c_a E_a(\tfrac{1}{4}), \qquad N(0_\pm )= c_s E_s(\tfrac{1}{4}+\tfrac{\tau }{2}) \pm c_a E_a(\tfrac{1}{4}+\tfrac{\tau }{2}), \end{aligned}$$

with $E_s(\frac{1}{4})\ne 0$ for all $\tilde{\Lambda }\in \mathbb {R}$ and $E_a(\frac{1}{4})\ne 0$ for all $\tilde{\Lambda }\ne \frac{1}{2} \pmod {1}$. Moreover, *N* will satisfy (3.18) if and only if $c_a=0$.

Note that in the special case $\tilde{\Lambda }=0 \pmod {1}$ we have (up to constants):

$$\begin{aligned} N_s((k,\pm ))=1, \qquad N_a((k,\pm ))= \frac{k^2+ c^2}{\pm w(k)}. \end{aligned}$$

In the case $\tilde{\Lambda }=\frac{1}{2} \pmod {1}$ we have (again up to constants):

$$\begin{aligned} N_s((k,\pm ))= \frac{k}{\sqrt{k^2+a^2}}, \qquad N_a((k,\pm ))= \frac{1}{\sqrt{k^2+a^2}}, \end{aligned}$$

where the root has the branch cut along $[-\mathrm {i}a,\mathrm {i}a]$.

Returning to our original problem we have shown:

#### Lemma 3.10

The function

$$\begin{aligned} m^{mod }(k) = \frac{\tilde{\gamma }(k)}{N_s(\infty _+)} \big (N_s((k,+)),N_s((k,-))\big ) \end{aligned}$$

is the unique vector-valued function which is holomorphic in the domain $\mathbb {C}\setminus [\mathrm {i}c, -\mathrm {i}c]$, has square integrable boundary values, and satisfies the jump condition (3.1), the symmetry condition (3.3) and the normalization condition (3.4).

Specifically, $m^{\text {mod}}(k)$ is continuous up to the boundary except at points of the set $\mathcal G:=\{ \mathrm {i}c, \mathrm {i}a, -\mathrm {i}a, -\mathrm {i}c\}$ where it has at most a fourth root singularity: $m^{\text {mod}}(k)= O((k-\kappa )^{-1/4}))$, $k\rightarrow \kappa$.

As shown previously in [9] and [10], the $m^{\text {mod}}(k)$ thus defined gives rise via Theorem 3.5 to an Its–Matveev solution (2.4) related to the elliptic Riemann surface $\hat{X}$.

## Matrix-valued solutions

In the framework of the nonlinear steepest descent analysis one usually needs to construct a matrix-valued R–H solution which is invertible. This is a necessary step to arrive at a small-norm R–H problem which can be solved via a Neumann series. However, while many integrable wave equations like the modified KdV equation [6, 16] or the nonlinear Schrödinger equation [7] have a matrix-valued R–H formulation, this is not the case for the KdV equation.

Recall that the model matrix-valued R–H problem related to the KdV equation has a jump matrix satisfying (see [9, 14])

4.1 $$\begin{aligned} v^{\text {mod}}(-k) = \sigma _1 (v^{\text {mod}}(k))^{-1} \sigma _1, \qquad \det v(k) = 1, \quad k \in [\mathrm {i}c, -\mathrm {i}c]. \end{aligned}$$

For the corresponding holomorphic matrix-valued solution $M^{\text {mod}}(k)$, one would then require

4.2 $$\begin{aligned} \lim _{k \rightarrow \infty } M^{\text {mod}}(k) = \begin{pmatrix} 1 &{} 0 \\ 0 &{} 1 \end{pmatrix}. \end{aligned}$$

Moreover, given holomorphicity of $M^{\text {mod}}(k)$, we can derive from (4.1) and (4.2):

4.3 $$\begin{aligned} M^{\text {mod}}(-k) = \sigma _1 M^{\text {mod}}(k) \sigma _1, \qquad \det M^{\text {mod}}(k) \equiv 1. \end{aligned}$$

Note that (4.3) implies that $M^{\text {mod}}(k)$ must have the form

$$\begin{aligned} M^{\text {mod}}(k) = \begin{pmatrix} \tilde{\alpha }(k) &{} \tilde{\beta }(-k) \\ \tilde{\beta }(k) &{} \tilde{\alpha }(-k) \end{pmatrix} \end{aligned}$$

and that $(1, 1)M^{\text {mod}}(k)$ will satisfy the symmetry condition (3.3).

As a first attempt of writing down an invertible matrix-valued model solution with at most fourth root singularities near the points $\kappa \in \mathcal G$ (c.f. Corollary 3.9), we start with the function

$$\begin{aligned} M^{\text {mod}}_1(k) = \tilde{\gamma }(k) \begin{pmatrix}\alpha _1(k) &{} \beta _1(-k)\\ \beta _1(k) &{} \alpha _1(-k)\end{pmatrix} \end{aligned}$$

where

$$\begin{aligned} \alpha _1(k)&= \frac{1}{2} \big ( N_a(\infty _+) N_s(k) + N_s(\infty _+) N_a(k) \big ), \\ \beta _1(k)&= \frac{1}{2} \big ( N_a(\infty _+) N_s(k) - N_s(\infty _+) N_a(k) \big ). \end{aligned}$$

Note that $M^{\text {mod}}_1(k)$ satisfies the symmetry condition in (4.3) and satisfies the normalization (4.2) up to a multiplicative factor. In fact we have

$$\begin{aligned} \lim _{k\rightarrow \infty }M^{\text {mod}}_1(k) = N_s(\infty _+) N_a(\infty _+) \begin{pmatrix} 1 &{} 0\\ 0 &{} 1\end{pmatrix}, \end{aligned}$$

with

$$\begin{aligned} \det M^{\text {mod}}_1(k)= N_s(\infty _+)^2 N_a(\infty _+)^2. \end{aligned}$$

The problem here is that the prefactor vanishes for $\tilde{\Lambda }= \frac{1}{2}\pmod 1$ as $N_a(\infty _+) = 0$, hence $N_a(k)$ is a vanishing solution of the model problem for these values $\tilde{\Lambda }$. It follows that we cannot enforce the normalization (4.2) for all $\tilde{\Lambda }$. In particular, $M^{\text {mod}}_1(k)$ is not invertible for these values of $\tilde{\Lambda }$. The relation to $m^{\text {mod}}(k)$ is given through

$$\begin{aligned} m^{\text {mod}}(k) = \frac{1}{N_s(\infty _+) N_a(\infty _+)} (1, 1) M^{\text {mod}}_1(k). \end{aligned}$$

where one needs to use the rule of l’Hôspital for $\tilde{\Lambda }= \frac{1}{2} \pmod 1$.

To obtain an invertible matrix-valued model solution also for $\tilde{\Lambda }= \frac{1}{2}\pmod 1$ we will have to give up on holomorphicity and introduce poles. To be precise, we will move the poles of the anti-symmetric solution to $\check{p}_{1,2} = \pm \frac{1}{4} + \frac{\tau }{2}$, which corresponds to a pole at $(0, \pm )$ on *X*. Following Sect. 3, this gives rise to an anti-symmetric solution of the form

4.4 $$\begin{aligned} \check{E}_a(z) = \frac{\theta _3(z+\frac{1}{4}-\frac{\tilde{\Lambda }}{2}-\frac{\tau }{2}|\tau )\theta _3(z-\frac{1}{4}-\frac{\tilde{\Lambda }}{2}+\frac{\tau }{2}|\tau )}{\theta _3(z+\frac{1}{4}|\tau )\theta _3(z-\frac{1}{4}|\tau )}, \qquad \check{N}_a(p) = \check{E}_a(A(p)). \end{aligned}$$

We can now define $M^{\text {mod}}_2(k)$ analogously to $M^{\text {mod}}_1(k)$, but substituting $\check{N}_a(k)$ for $N_a(k)$, and including the correct normalization at infinity for $\tilde{\Lambda }\not = 0 \pmod 1$:

$$\begin{aligned} M^{\text {mod}}_{2}(k) = \frac{\tilde{\gamma }(k)}{N_s(\infty _+)\check{N}_a(\infty _+)} \begin{pmatrix}\alpha _2(k) &{} \beta _2(-k)\\ \beta _2(k) &{} \alpha _2(-k)\end{pmatrix}, \qquad \tilde{\Lambda }\not = 0 \pmod 1, \end{aligned}$$

where

$$\begin{aligned} \alpha _2(k)= & {} \frac{1}{2} \big (\check{N}_a(\infty _+) N_s(k) + N_s(\infty _+) \check{N}_a(k) \big ), \\ \beta _2(k)= & {} \frac{1}{2} \big ( \check{N}_a(\infty _+) N_s(k) - N_s(\infty _+) \check{N}_a(k) \big ), \end{aligned}$$

Note that $N_s(\infty _+)\check{N}_a(\infty _+)\not = 0$ for $\tilde{\Lambda }\not = 0 \pmod 1$ and thus we have:

$$\begin{aligned} \lim _{k\rightarrow \infty } M^{\text {mod}}_2(k) = \begin{pmatrix} 1 &{} 0 \\ 0 &{} 1 \end{pmatrix}, \qquad \tilde{\Lambda }\not = 0 \pmod 1. \end{aligned}$$

Moreover, $\det M^{\text {mod}}_2(k)$ is an even meromorphic function with at most a simple pole at the origin, hence $\det M^{\text {mod}}_2(k) \equiv \det M^{\text {mod}}_2(\infty )= 1$. We do not define $M^{\text {mod}}_2(k)$ for $\tilde{\Lambda }= 0 \pmod 1$, which should not pose a problem in applications as the standard holomorphic matrix-valued solution $M^{\text {mod}}_1(k)$ can be constructed in this case.

It turns out that sacrificing holomorphicity, while retaining (4.2) and (4.3), is the most convenient way to deal with the ill-posedness of the holomorphic matrix-valued model problem for the KdV equation. Indeed, this was the strategy in [11, Sect. 6] and [13, Sect. 3.3]. Note however, that the anti-symmetric meromorphic vector solutions found [11] and [13] are not the same as given by (4.4). The reason is, that while we assumed that $\check{N}_a(p)=\check{E}_a(A(p))$ has only poles at $0_\pm$, this condition was more strict than necessary, as we could still allow for singularities at $\pm \mathrm {i}a$, as is the case for $m^{\text {mod}}(k)$. Indeed, $m^{\text {mod}}(k)/k$ has a pole at the origin and is an anti-symmetric solution to the vanishing problem where solutions are required to vanish at infinity. Hence, there is no chance for uniqueness if we allow for poles at 0 and fourth root singularities at $\pm \mathrm {i}a$. Moreover for $\tilde{\Lambda }= 0 \pmod 1$, $m^{\text {mod}}(k)/k$ has no singularities at $\pm \mathrm {i}a$, and hence by our uniqueness Lemma 3.8 must coincide with the solution generated by $\check{N}_a(p)$.

Interestingly, any anti-symmetric solution with a simple pole at the origin and fourth root singularities at $\pm \mathrm {i}a$, which is normalized to $(-1 \ \ 1)$ at infinity, is adequate for the analysis performed in [11]. The reason is that the pole cancellation in the final step of the nonlinear steepest descent analysis is due to the underlying symmetry class, rather than the exact form of the second vector-valued solution (see Lemma 6.4 in [11]).

On a more geometric side, it is interesting to observe that while the model R–H solution $m^\text {mod}(k)$ can be conveniently defined on a ’smaller’ Riemann surface $\hat{X}$ as in (3.26), for the nonlinear steepest descent analysis it seems to be necessary to consider the Riemann surface *X* which is a double covering of $\hat{X}$.

## Data Availability

Data sharing not applicable to this article as no datasets were generated or analysed.
